# Supplementary material for: CD44 Depletion in Glioblastoma Cells Suppresses Growth and Stemness and Induces Senescence
Source: Cancers (Basel). 2022 Jul 31;14(15):3747. doi: 10.3390/cancers14153747 (PMC9367353; doi:10.3390/cancers14153747)
Supplement: Supplementary file 1 [file cancers-14-03747-s001.zip › Proofreading supplementary.pdf]

Supplementary Materials: CD44 Depletion in Glioblastoma Cells Suppresses Growth and Stemness and Induces Senescence

Constantinos Kolliopoulos, Mohamad Moustafa Ali, Casimiro Castillejo-Lopez, Carl-Henrik Heldin and Paraskevi Heldin

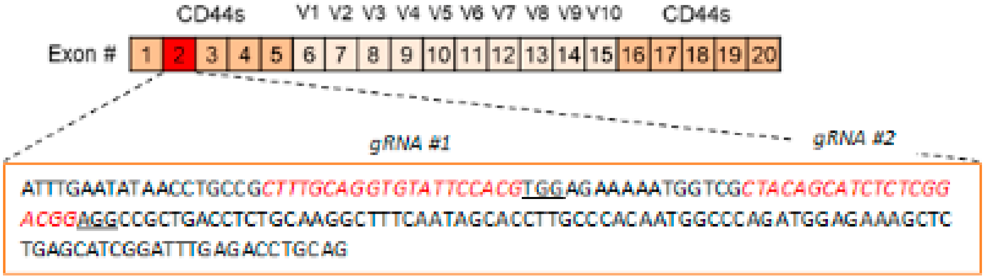

Figure S1. Representation of gRNAs targeting CD44. Coding region of *CD44* is shown; constant exons generating CD44s are depicted with orange and variable (V) exons giving rise to CD44v are demonstrated with light orange. Exon #2 (red), was targeted with two gRNAs represented in italics followed by the PAM sequence, which is underlined. #, number.

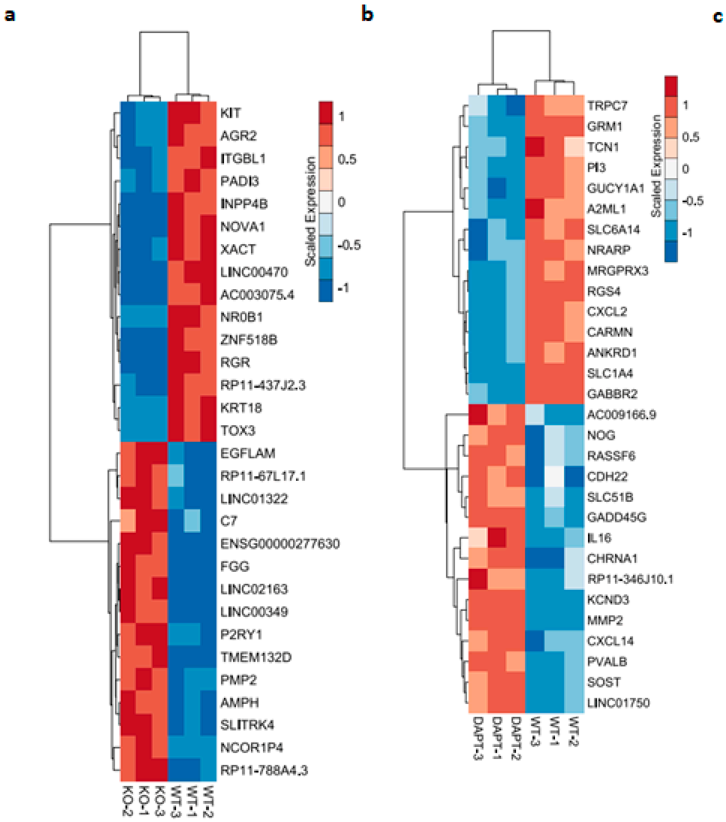

c

Common genes	
Down-	Up-
AGR2	JPH1
RP11-437J2.3	ARHGAP42
KIT	MUC20
LGI1	RASGEF1C
ELF3	HECW1
SLC39A12	RXFP4
LINC02302	GLCCI1
SCNN1A	BMPER
AGR3	RP11-275H4.1
ADCYAP1R1	EGR2

Figure S2. Inhibition of CD44 cleavage partially reproduces CD44 depletion in sphere-like conditions. (**a**,**b**), Heatmaps of relative expression values of the top 15 down- or upregulated genes of CD44 KO (**a**) or parental U251MG cells treated with the γ-secretase inhibitor DAPT (**b**) compared to control U251MG cells are shown. (**c**), Table with the top 10 common down or upregulated genes when parental U251MG cells are compared with either parental U251MG DAPT-treated or CD44 KO cells. Cells were grown in low attachment conditions.

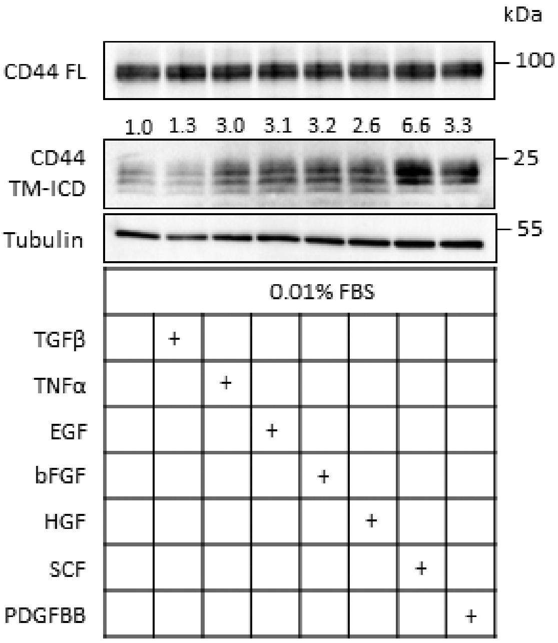

Figure S3. SCF and PDGF-BB potently induce CD44 cleavage in U251MG. Immunoblotting analysis of full-length (FL) and cleavage products (transmembrane and intracellular domains, TM-ICD) of CD44 in U251MG cells, after treatment for 24 h with the indicated growth factors/cytokines (all at a final concentration of 10 ng/mL). Uncropped immunoblots are shown in Figure S10. +, presence of the indicated factor.

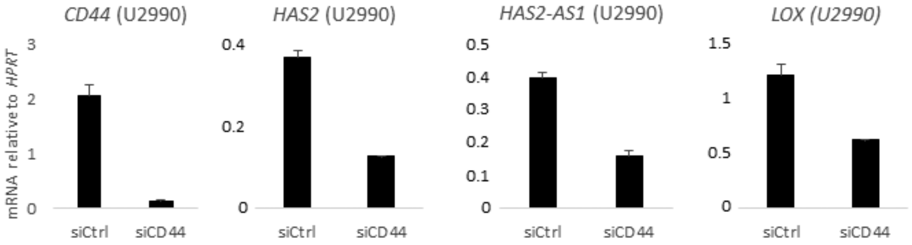

Figure S4. siRNA-mediated CD44 knockdown recapitulates CD44 knockout-mediated gene expression in U2990 cells. mRNA levels of *CD44, HAS2, HAS2-AS1* and *LOX* mRNAs were quantified by real-time qPCR after normalization to *TBP* in U2990 cells transfected with an siRNA targeting CD44 or with non-targeting scrambled siRNAs. Cells were grown in sphere-like conditions. One representative experiment out of two is demonstrated.

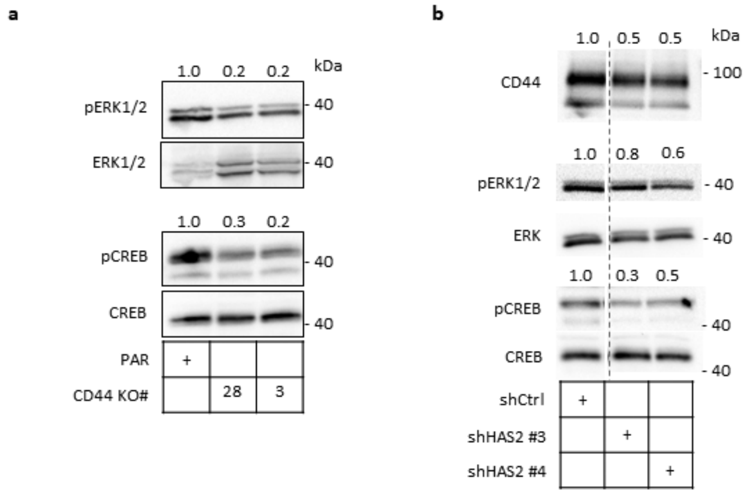

Figure S5. Perturbation of ERK1/2 MAPK and CREB signaling pathways in CD44 KO cells or U251MG cells stably transfected with shHAS2. (**a**,**b**), Immunoblotting analysis of full-length (FL) of

CD44, p-ERK1/2, ERK1/2, p-CREB and CREB levels in either CD44 KO clones (**a**) or shHAS2 stably transfected (**b**) U251MG cells, when cultured in non-adherent condition. Quantification of the immunoreactive bands was performed by ImageJ after normalizing to total levels of the corresponding phosphorylated proteins or total ERK1/2 levels in the case of CD44 (**b**), and shown as fold difference compared to the respective control. Uncropped immunoblots are shown in Figure S11.

Fig. 1a

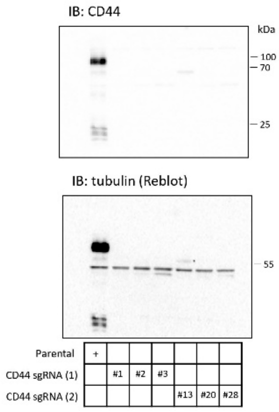

Figure S6. Uncropped Western Blot images for Figure 1a. #, number; +, presence of the indicated cell line.

Fig. 1e

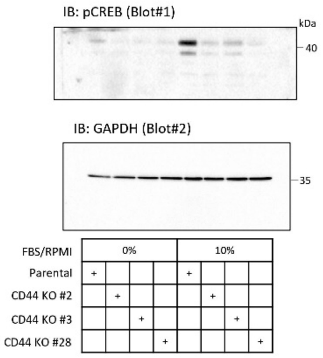

Figure S7. Uncropped Western Blot images for Figure 1e. +, presence of the indicated cell line.

Fig. 1g

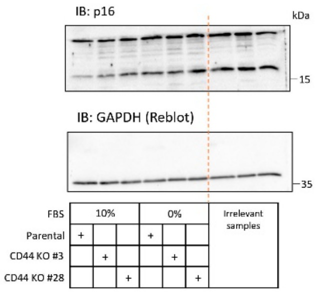

Figure S8. Uncropped Western Blot images for Figure 1g. +, presence of the indicated cell line.

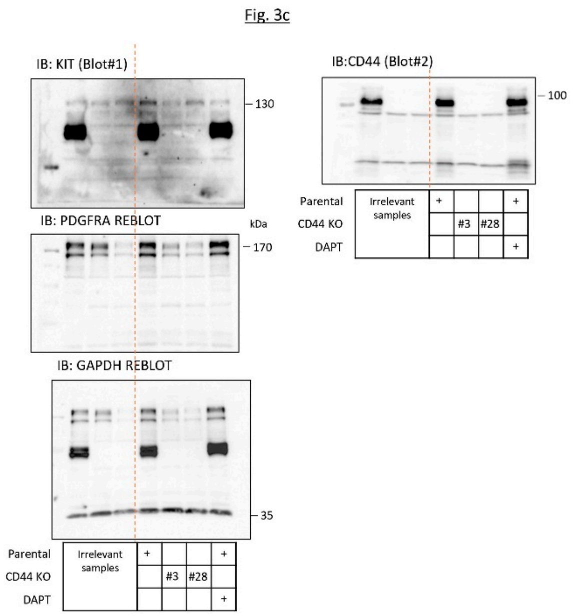

Figure S9. Uncropped Western Blot images for Figure 3c. #, number; +, presence of the indicated cell line or compound.

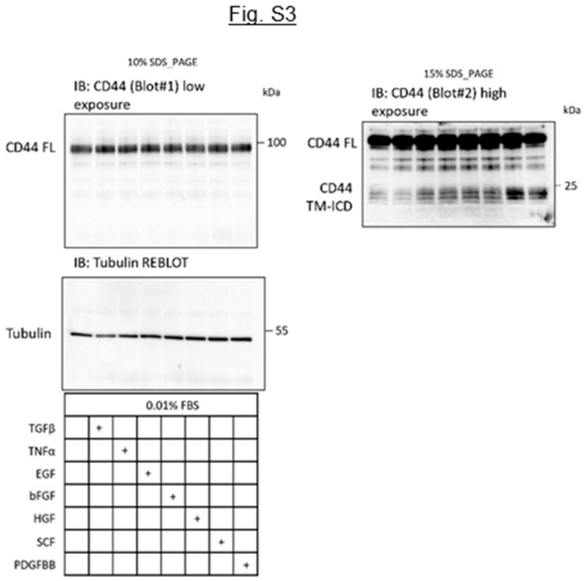

Figure S10. Uncropped Western Blot images for Figure S3. +, presence of the indicated factor.

Fig. S5

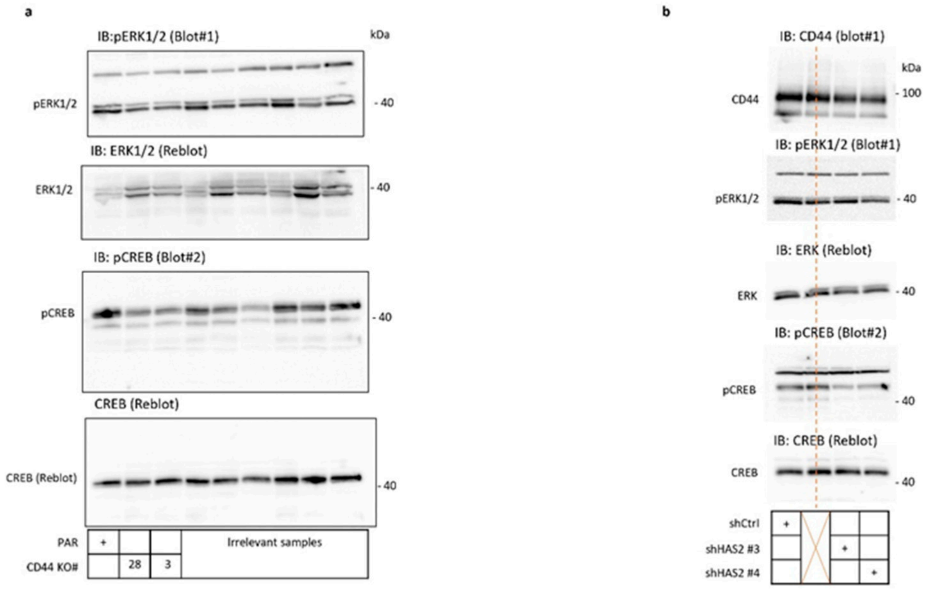

Figure S11. Uncropped Western Blot images for Figure S5. +, presence of the indicated factor.

Table S1. Specific gene signatures (hallmarks) CD44 KO vs Parental and DAPT vs Parental.

Table S2. Complete Filtered_DEGs_and_Common_DEGs_All_samples.

Table S3. Complete Hallmarks_Lists_CD44KO_vs_Parental.

Table S4. Complete Hallmarks_DAPT_vs_Parental.

Table S1. is provided separately attached as a PDF file.

Table S2–S4. are provided separately attached as Excel files.
