# Supplementary material for: CD44 Depletion in Glioblastoma Cells Suppresses Growth and Stemness and Induces Senescence
Source: Cancers (Basel). 2022 Jul 31;14(15):3747. doi: 10.3390/cancers14153747 (PMC9367353; doi:10.3390/cancers14153747)
Supplement: Supplementary file 1 [file cancers-14-03747-s001.zip › Table S1.pdf]

Specific gene signatures (hallmarks) CD44 KO vs Parental and DAPT vs Parental

Parental vs CD44 KO		ES	NES	NOMp
1	Estrogen Receptor Early	-0.58	-1.81	0.000
2	Hypoxia	-0.57	-1.80	0.000
3	Estrogen Repsonse Late	-0.54	-1.68	0.000
4	Reactive Oxygen Species	-0.61	-1.57	0.007
5	Glycolysis	-0.50	-1.57	0.000
6	IL6/JAK/STAT3 Signaling	-0.54	-1.52	0.005
7	G2M Checkpoint	-0.48	-1.51	0.000
8	Epithelial Mesenchymal Transition	-0.47	-1.46	0.002

Parental vs DAPT		ES	NES	NOMp
1	E2F Targets	-0.81	-2.74	0.000
2	G2M Checkpoint	-0.77	-2.63	0.000
3	MTORC1 Signaling	-0.69	-2.32	0.000
4	Glycolysis	-0.66	-2.25	0.000
5	Hypoxia	-0.65	-2.20	0.000
6	Myc Targets	-0.63	-2.14	0.000
7	TNFα Signaling via NFkB	-0.61	-2.08	0.000
8	Mitotic Spindle	-0.59	-2.00	0.000
9	Estrogen Repsonse Late	-0.58	-1.98	0.000
10	Oxidative Phosphorylation	-0.58	-1.96	0.000
11	UV Response Up	-0.58	-1.89	0.000
12	Cholesterol Homeostasis	-0.63	-1.88	0.000
13	Inflammatory Response	-0.55	-1.87	0.000
14	Interferon α Response	-0.60	-1.84	0.000
15	Estrogen Repsonse Early	-0.52	-1.77	0.000
16	UV Response Down	-0.54	-1.76	0.000
17	IL6/JAK/STAT3 Signaling	-0.56	-1.73	0.000
18	Unfolded Protein Response	-0.55	-1.72	0.000
19	IL2/STAT5 Signaling	-0.50	-1.70	0.000
20	Angiogenesis	-0.65	-1.69	0.006
21	Androgen Response	-0.55	-1.69	0.000
22	Adipogenesis	-0.50	-1.68	0.000
23	Reactive Oxygen Species	-0.60	-1.67	0.000

Table S1. Gene signatures in U251MG cells grown in spheres. Specific gene signatures (hallmarks) upon CD44 KO in U251MG cells or treatment of parental U251MG cells with DAPT, compared with parental control cells, are shown.
