# Supplementary figures and images for: CD44 Depletion in Glioblastoma Cells Suppresses Growth and Stemness and Induces Senescence
Source: Cancers (Basel). 2022 Jul 31;14(15):3747. doi: 10.3390/cancers14153747 (PMC9367353; doi:10.3390/cancers14153747)

Fig. 1a

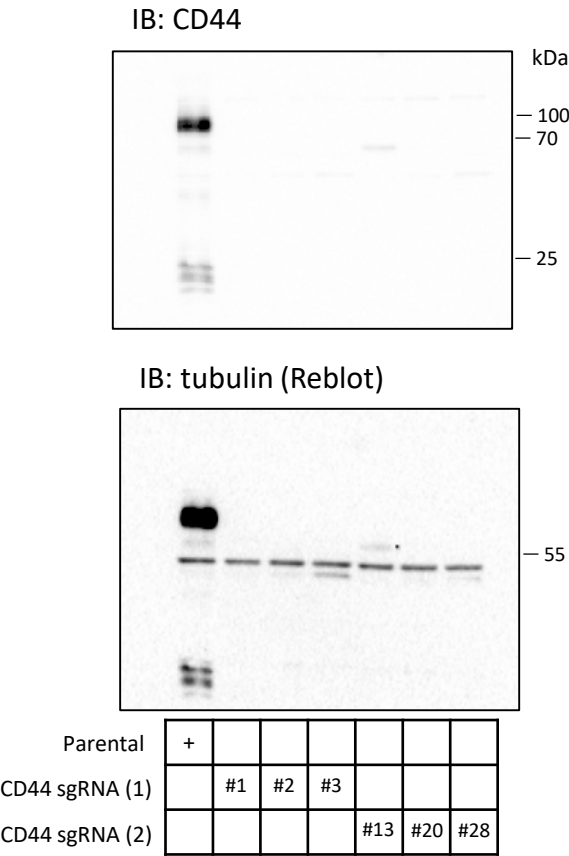

Fig. 1e

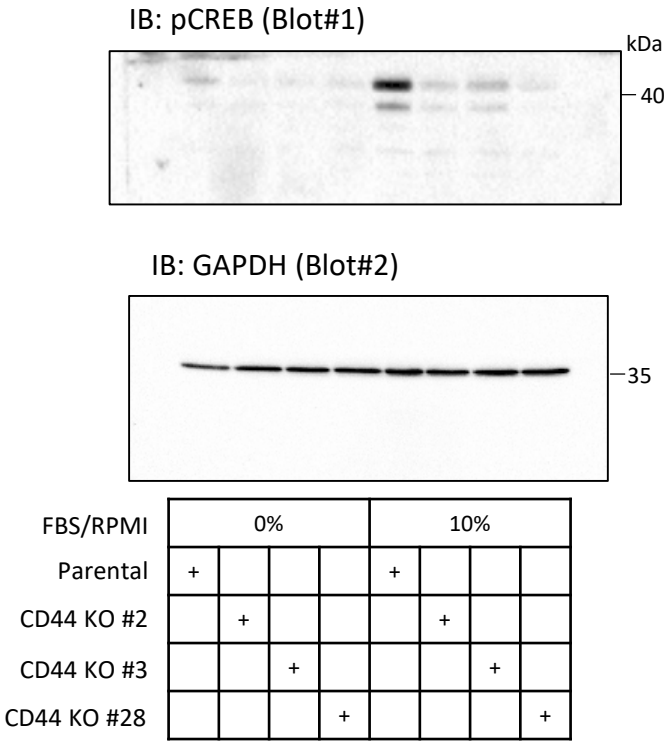

Fig. 1g

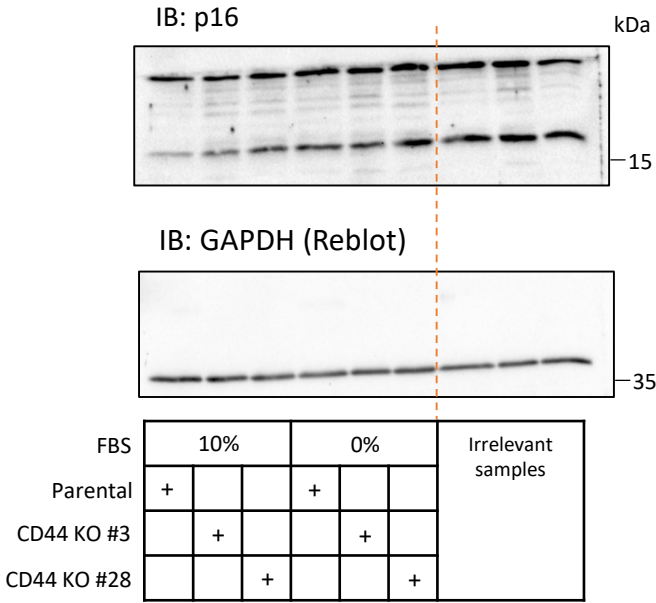

Fig. 3c

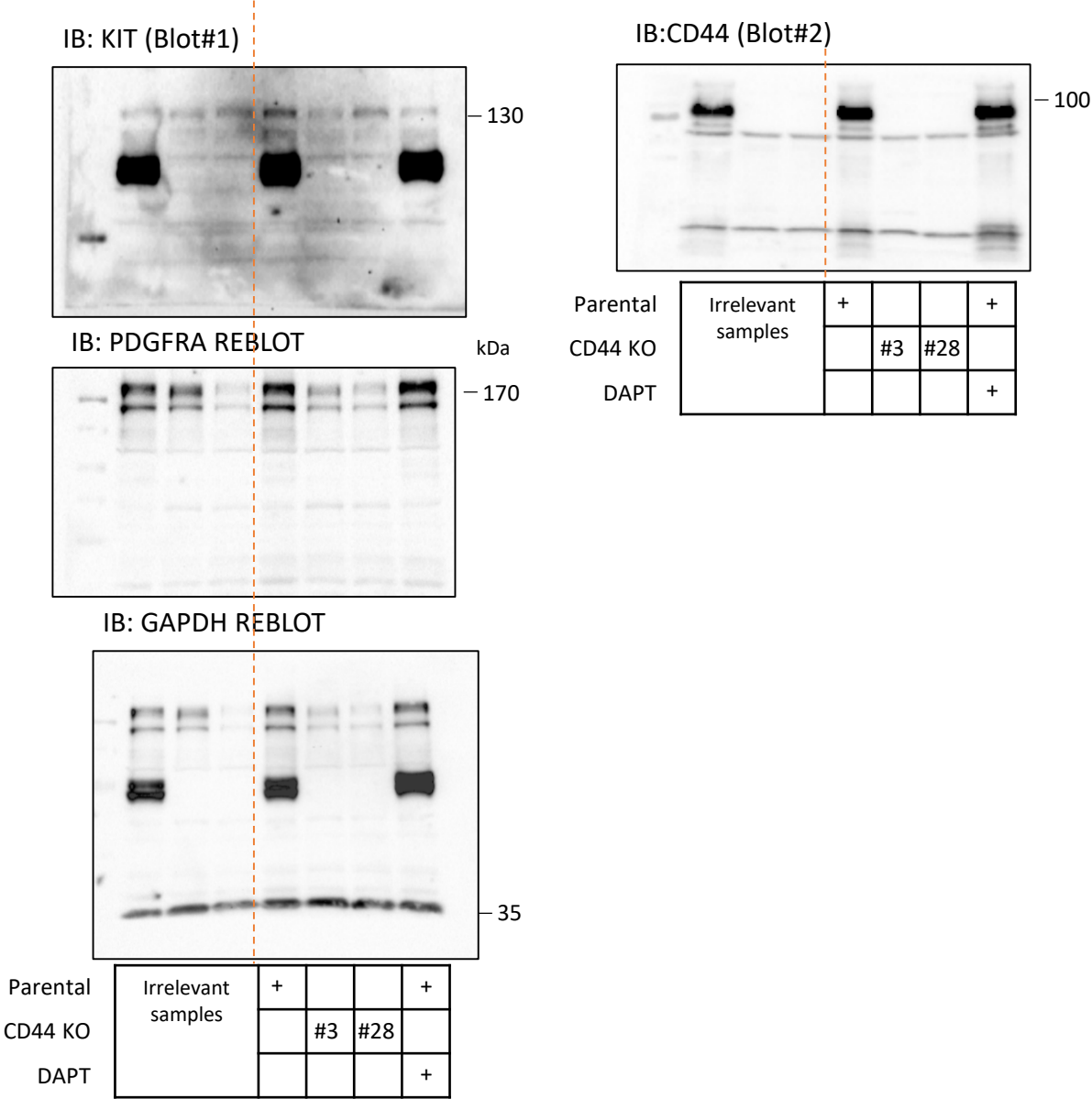

Fig. S4

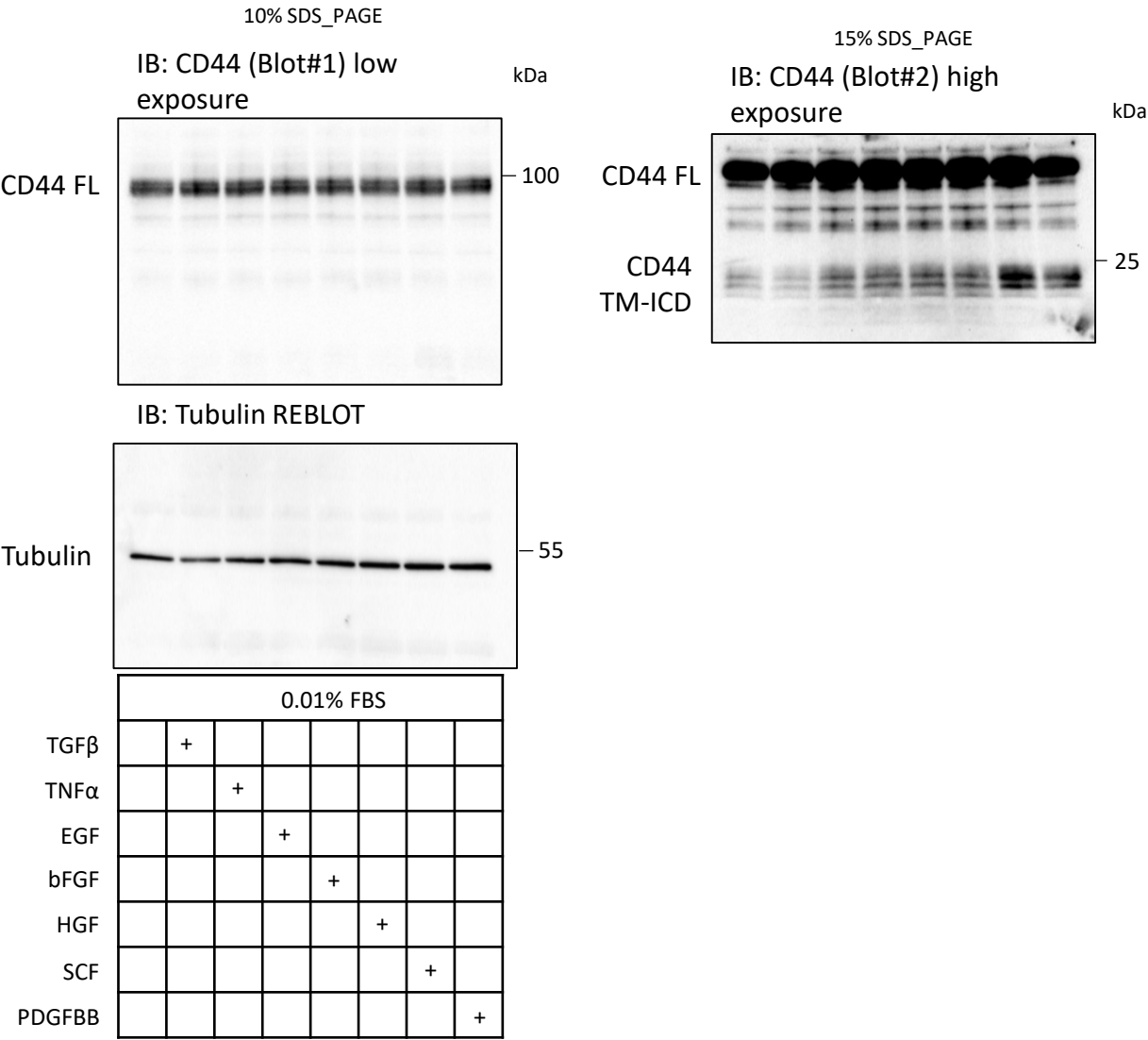

Fig. S6

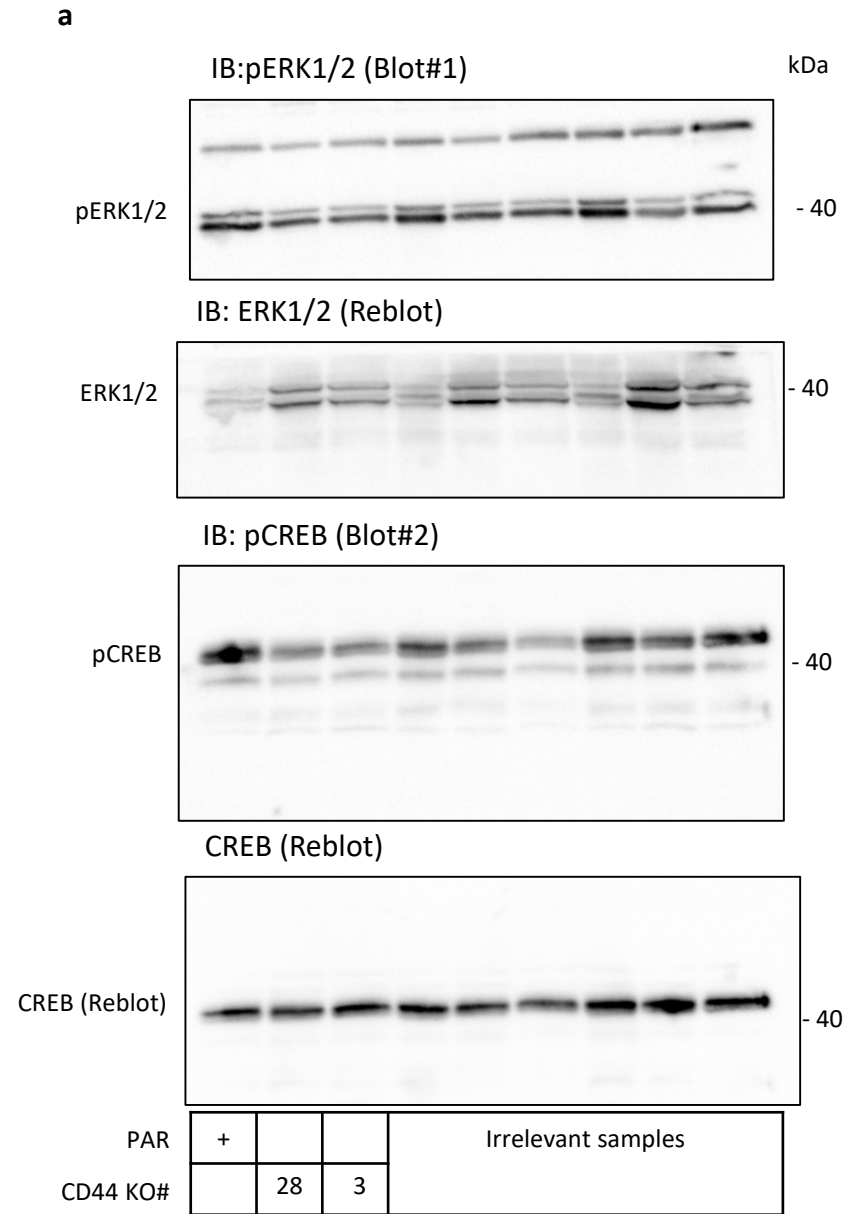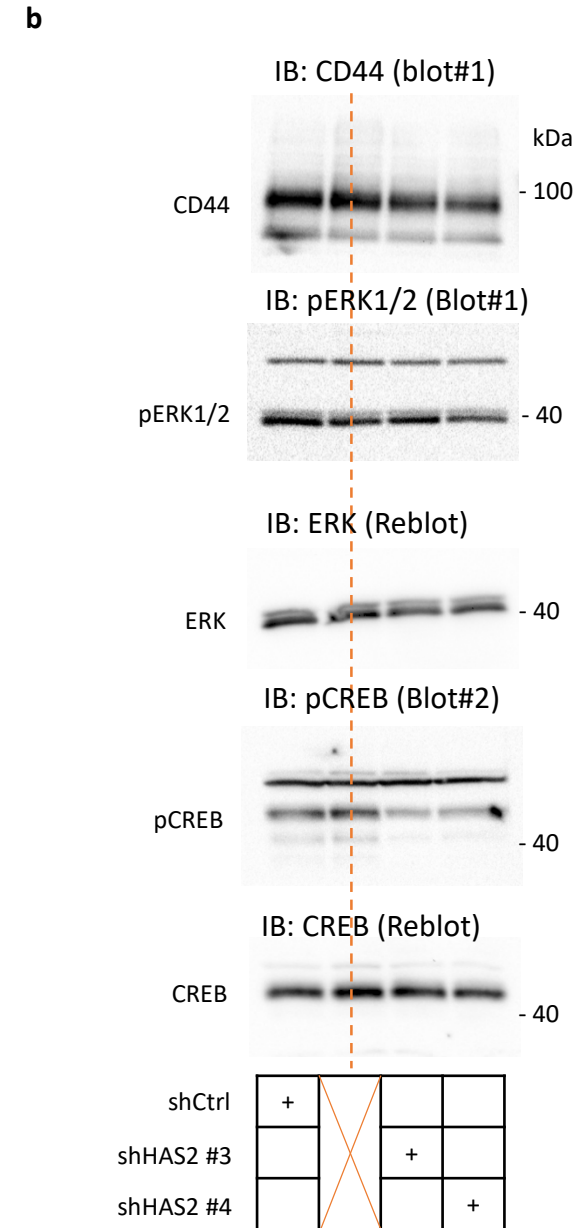

Supplement: Supplementary file 1 [file cancers-14-03747-s001.zip › cancers-1805721-original-images.pdf]
